# Association of *PRPS1* Mutations with Disease Phenotypes

**DOI:** 10.1155/2015/127013

**Published:** 2015-05-24

**Authors:** Rahul Mittal, Kunal Patel, Jeenu Mittal, Brandon Chan, Denise Yan, M'hamed Grati, Xue Zhong Liu

**Affiliations:** ^1^Department of Otolaryngology, University of Miami Miller School of Medicine, Miami, FL 33136, USA; ^2^Department of Human Genetics, University of Miami Miller School of Medicine, Miami, FL 33136, USA; ^3^Department of Biochemistry, University of Miami Miller School of Medicine, Miami, FL 33136, USA

## Abstract

*Phosphoribosylpyrophosphate synthetase 1* (*PRPS1*) codes for PRS-I enzyme that catalyzes the first step of nucleotide synthesis. *PRPS1* gene mutations have been implicated in a number of human diseases. Recently, new mutations in *PRPS1* have been identified that have been associated with novel phenotypes like diabetes insipidus expanding the spectrum of *PRPS1*-related diseases. The purpose of this review is to evaluate current literature on *PRPS1*-related syndromes and summarize potential therapies. The overexpression of *PRPS1* results in PRS-I superactivity resulting in purine overproduction. Patients with PRS-I superactivity demonstrate uric acid overproduction, hypotonia, ataxia, neurodevelopment abnormalities, and postlingual hearing impairment. On the other hand, decreased activity leads to X-linked nonsyndromic sensorineural deafness (DFNX-2), Charcot-Marie-Tooth disease-5 (CMTX5), and Arts syndrome depending on the residual activity of PRS-I. Mild PRS-I deficiency (DFNX-2) results in non-syndromic progressive hearing loss whereas moderate PRS-I deficiency (CMTX5) and severe PRS-I deficiency (Arts syndrome) present with peripheral or optic neuropathy, prelingual progressive sensorineural hearing loss, and central nervous system impairment. Currently, purine replacement via S-adenosylmethionine (SAM) supplementation in patients with Arts syndrome appears to improve their condition. This suggests that SAM supplementation can alleviate symptoms of *PRPS1* deficient patients and open new avenues of therapeutic intervention.

## 1. Introduction

Human phosphoribosylpyrophosphate synthetase (PRS) is one of the crucial enzymes in the* de novo* synthesis and salvage of purines and biosynthesis of pyrimidine and pyridine nucleotides [1–4].* PRPS1, *transcriptionally regulated by microRNA-376 [5], codes for PRS-I, which catalyzes the synthesis of phosphoribosylpyrophosphate (PRPP) from adenosine triphosphate (ATP) and ribose-5-phophate (R5P) [4, 6]. The enzymatic activity of PRS-I is activated by inorganic phosphate and Mg^2+^ but can be allosterically inhibited by adenosine diphosphate (ADP) and purines [2–4, 7, 8]. This enzyme occurs in three isoforms (PRS-I, PRS-II, and PRS-1L1) encoded by the genes* PRPS1*,* PRPS2*, and* PRPS1L1, *respectively; these genes are highly conserved among vertebrates ranging from zebra fish to humans [8].* PRPS1* (MIM 311850) and* PRPS2* (MIM 311860) are mapped on opposite arms of the X-chromosome and are expressed in many different tissues (locus Xq22-q24 and Xq22.3-P22.3, resp.).* PRPS1L1* (MIM611566) is located on an autosomal gene and is expressed significantly in the testis [2]. To date, mutations in the* PRPS2* and* PRPS1L1 *have not been known to cause disease.

**Figure 1:**
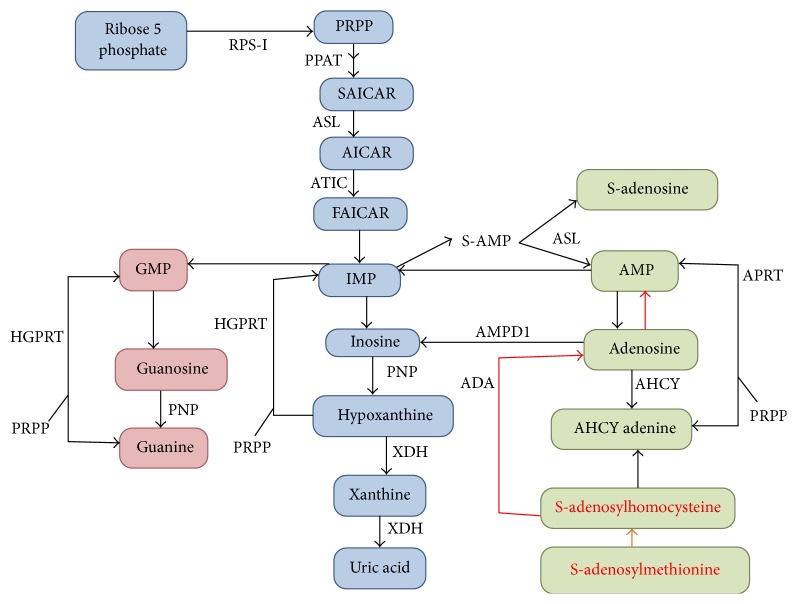
A schematic representation of purine metabolic pathway. S-adenosylmethionine (SAM) can replenish ATP and GTP independently of PRPP by direct conversion into adenine via the polyamine pathway (indicated by red color). Alternatively, methyltransferases transform SAM into S-adenosylhomocysteine which can be then converted into adenosine and adenine.

In the energy intensive synthesis of purines, PRPP is a substrate for PRPP amidotransferase, the first and rate-limiting step of* de novo* purine synthesis, ultimately producing purine nucleotides guanine triphosphate (GTP) and adenine triphosphate (ATP) [4, 6]. PRPP is also used as a substrate for the “salvage” of purine bases, responsible for converting adenine to adenosine monophosphate (AMP) via adenine phosphoribosyl transferase and converting inosine monophosphate (IMP) to guanine monophosphate (GMP) via hypoxanthine guanine phosphoribosyl transferase (Figure 1) [4, 6]. In the pyrimidine nucleotide synthesis, PRPP is an important cofactor for uridine monophosphate synthetase, which assists in converting orotic acid to UMP, the precursor for all pyrimidine nucleotides [6]. Lastly, PRPP plays a key role in pyridine nucleotide synthesis by nicotinate phosphoribosyl transferase (NAPRT) and nicotinamide phosphoribosyl transferase (NAMPT), which form nicotinamide adenine dinucleotide (NAD) and nicotinamide adenine dinucleotide phosphate (NADP), respectively. Thus, mutations in the* PRPS1 *can have a profound impact on several vital cell processes, such as nucleic acid synthesis, energy metabolism, and cellular signaling.

*PRPS1* is expressed in several different tissues [9] including prenatal and postnatal inner ear organs: cochlea, vestibular organs, and utricle [5]. The transcript levels of* PRPS1* are regulated by* mir-376*. MicroRNAs (miRNAs) are short ~22 nucleotide RNA sequences that bind to complementary sequences in the 3′ UTR of multiple target mRNAs, thereby inhibiting protein synthesis or causing mRNA degradation [10–12]. Our previous study demonstrated that miR-376a-3p, b-3p, c-3p are present in mouse embryonic inner ears and intensive expression of miR-376a-3p/b-3p was detected in the sensory epithelia and ganglia of both auditory and vestibular portions of the inner ear [8]. In adult inner ear, the expression of miR-376a-3p/b-3p is restricted within ganglion neurons of auditory and vestibular systems as well as the cells in the stria vascularis [8]. Earlier studies from our laboratory also showed continuous expression of the* PRPS1* gene in cochlear and vestibular hair cells as well as postnatal expression in the spiral ganglia by* in situ* hybridization (ISH) [5]. At embryonic day 18.5,* PRPS1* was found to be expressed in the following tissues: utricle, crista, and cochlear hair cells, Claudius cells, and the greater epithelial ridge but not in cochlear supporting cells [5]. Postnatal* PRPS1* expression was observed in the spiral ganglion cells and the hair cells in the organ of corti [5]. Thus,* PRPS1* expression plays a role in fetal inner ear development, and mutations to the* PRPS1* gene or to miRNA-376 may result in abnormal organ development.

Missense mutations in the* PRPS1 *gene have resulted in the classification of four distinct nonsyndromic and syndromic forms of hearing loss: PRS-I superactivity (MIM 300661), X-linked nonsyndromic sensorineural hearing loss (DFN-2) (MIM 304500), Charcot-Marie-Tooth disease-5 (CMTX5) (MIM 311070), and Arts syndrome (MIM 301835) (Table 1). Until recently, 15* PRPS1* mutations have been identified: 2 in Arts syndrome, 2 in CMTX5 syndrome, 7 in* PRPS1* superactivity, and 4 in DFNX-2 (Table 1). However, several novel mutations in the* PRPS1 *have been recently published (Table 1) suggesting that these disorders exist as a phenotypic continuum of* PRPS1*-related diseases.

## 2. *PRPS1* Structure

The enzymatic active unit is a hexamer, consisting of three homodimers arranged in a propeller-like shape [1, 2, 17, 24]. Each of the homodimers has an active site and two regulatory allosteric sites, I and II. The active site allows for ATP and R5P to bind at the junction of two domains within one homodimer. Allosteric site I is located at the interface of the three homodimers and allosteric site II is located at the interface of two monomers within one homodimer [1, 2, 17, 24]. Mutations in the* PRPS1* can either result in a gain of function with increased expression,* PRPS1* superactivity, or result in a loss of function with a decreased expression, DFN-2, CMTX5, and Arts syndrome.

## 3. PRS-I Superactivity

PRS-I superactivity is an X-linked disorder classically associated with hyperuricemia and hyperuricosuria resulting in childhood gout [25]. PRS-I superactivity has been demonstrated to exhibit a wide range of phenotypes. Patients with the milder phenotype of PRS-I superactivity, the more common phenotype, present an overproduction of purines without any neuropathy. Uric acid crystalluria or urinary stones are commonly the first clinical finding followed by gout arthritis if the uric acid levels are not controlled. Patients with the more severe phenotype exhibit similar early-childhood gout arthritis that is also accompanied with a combination of neurodevelopment abnormalities, hypotonia, ataxia, and sensorineural hearing loss [17, 25].

Two different mechanisms have been proposed that can cause PRS-I superactivity: a gain-of-function point mutations in the open reading frame (ORF) of* PRPS1 *that results in an altered regulatory site, and an increased expression of* PRPS1* that has normal kinetic enzyme properties [1, 13, 15, 25–34]. There have been more than 30 patients reported to have a severe form of* PRPS1 *superactivity. Seven missense mutations in the* PRPS *have been extracted from these patients [1, 3, 11, 12].

Two of the seven missense mutations (c.154G>C (p.D52H) and c.385C>A (p.L129I)) causing PRS-I superactivity have been found to disrupt one of the two allosteric sites, therefore inhibiting feedback inhibition [1, 35]. The p.D52H mutation results in the destabilization of the local structure around Asp52 therefore affecting allosteric site I [1]. The p.L129I mutation results in steric hindrance with the protein backbone at Ala131 and Ile134 therefore destabilizing the local structure, disrupting the allosteric site II [1].

The five remaining missense mutations (c.341A>G (p.N114S), c.547G>C (p.D182H), c.569C>T (p.A189V), c.578A>T (p.H192L), and c.579C>G (p.H192Q)) are located at the interface of the homodimer interface [1, 14, 16]. These missense mutations disrupt the entire homodimer itself and both allosteric sites. Allosteric site II is primarily affected due to the central location of these point mutations [1].

The point mutation c.424G>C (p.V142L), a novel* PRPS1* mutation resulting in an increased activity of PRS-I, was recently found in a young boy with the classic findings of uric acid overproduction but with no gout arthritis, as is typically noted in patients with PRS-I superactivity [17]. The patient also experienced developmental delay, hypotonia, and bilateral hearing loss similar to the severe form of PRS-I superactivity [17]. Moreover, the patient complained of recurrent respiratory infections, myopia, glaucoma, and motor neuropathy; these manifestations mostly occur in Arts syndrome (see below) [17]. Molecular modeling of this mutation suggests that the ATP binding site is affected in addition to the allosteric site II [17]. This novel mutation exhibits a phenotype with characteristics of both gain-of-function and loss-of-function mutations. This is the first missense mutation to connect the two extremes of the PRS-I-related disorders spectrum [17].

## 4. X-Linked Nonsyndromic Sensorineural Hearing Deafness (DFNX-2)

The mildest form of loss-of-function* PRPS1* activity results in DFNX-2, characterized by only having nonsyndromic hearing loss. These mutations can result in a wide spectrum of phenotypes of hearing loss: bilateral, moderate to profound, progressive or nonprogressive, and prelingual or postlingual [36, 37]. Some males showed early-onset (7–20 years of age) moderate hearing loss with an upward sloping audio profile, with low to middle frequency hearing impairment, but retained the ability to hear high frequency sounds, whereas other male patients exhibited profound hearing loss and a flat audio profile with a later onset [36, 37]. Female carriers can be normal or abnormal, but if they were abnormal, their hearing impairment was less profound than the affected males [16].

Thus far, four missense mutations have been identified in four separate families: c.193G>A (p.D65N), c.259G>A (p.A87T), c.869T>C (p.I290T), and c.916G>A (p.G306R) [1, 5]. None of the identified mutations have any major changes on the overall structure of the enzyme, which explains the mild phenotype of only nonsyndromic hearing loss. Using molecular modeling techniques the D65N mutation has been shown to affect the ATP binding site [1, 5]. Similar predictions have been made about the remaining three mutations and their effects on the overall structure. Compared to controls,* in vitro* enzymatic activity assays of affected patients' erythrocytes and cultured fibroblasts have a 44%-45% decrease in PRS-I activity [5].

## 5. Novel Mutations Bridging the Spectrum between DFNX-2 and CMTX5

Four new patients with novel mutations in the* PRSP1* have been identified that exhibit symptoms of DFNX-2: c.337G>T (p.All3S), c.343A>G (p.M115V), c.925G>T (p.V309F), and c.362C>G (p.A121G) [18, 19].

The p.A113S mutation, being the least severe of the four new mutations, only resulted in postlingual nonsyndromic hearing loss but was met with no signs of clinical or subclinical peripheral neuropathy [18]. This mutation is located at the trimer interface and is predicted, via molecular modeling, to destabilize the surrounding environment therefore affecting the ATP binding site [18].

The patients' p.M115V and p.V309F mutations resulted in postlingual nonsyndromic hearing loss with a downward sloping audio profile. In addition, these patients also showed signs of clinical and subclinical peripheral neuropathy. The mild neuropathy is predominately sensory based and is characterized by absence of deep tendon reflexes, parenthesis, and cramps; no muscle weakness of motor deficits was reported [18]. Both of these mutations are also located at the trimer interface and are predicted to destabilize the surrounding environment affecting both the ATP binding site and the allosteric site I. Lastly, the p.A121G mutation resulted in early-onset bilateral profound sensorineural hearing loss and peripheral neuropathy, resembling CMTX5 syndrome, but no optic atrophy was reported [19]. The peripheral neuropathy, compared to p.M115V and p.V309F, was both motor and sensory related; neurological exams revealed bilateral weakness and atrophy of distal muscles, prominent gait disturbances, lack of deep tendon reflexes, and severely impaired pain and temperature senses [19]. No molecular modeling was done on this mutation but based on* PRPS1's* primary sequence and the quaternary structure, it is expected to affect the catalytic site (ATP binding site and R5P binding site) thus reducing the enzymatic activity [19].

## 6. Charcot-Marie-Tooth Disease (CMTX5) or Rosenberg-Chutorian Syndrome

CMTX5 is an extremely rare X-linked genetic disorder with only two known missense mutations; however, due to the rarity of the disease, it may be underdiagnosed and underrecognized by pediatricians. This disorder is characterized by a “triad” of symptoms: peripheral neuropathy, early-onset (prelingual) bilateral profound sensorineural hearing loss, and optic neuropathy [20, 38]. Other reported symptoms include hypotonia, gait disturbances, and loss of deep tendon reflexes [1].

Thus far, two missense mutations have been identified: c.129A>C (p.E43D) and c.344T>C (p.M115T) [30]. Both of these mutations are involved in the* PRPS1* trimer interface and affect the ATP binding pocket [1, 3, 20]. Furthermore, p.M115T mutation is also predicted to interact with the allosteric site I [1, 20]. Compared to controls,* in vitro* enzymatic activity assays in affected patients' cultured fibroblasts have a 62% decrease in* PRPS1* activity [5].

## 7. Novel Mutations Bridging the Spectrum between Arts Syndrome and CMTX5

A new family with a novel* PRPS1* missense mutation, c.830A>C (p.Q277P), has been reported with features resembling both CMTX5 and Arts syndrome. Similar to CMTX5, the patients exhibited a “triad”: prelingual hearing loss, optic atrophy with early teen onset, and severe sensorineural motor neuropathy. These symptoms overlap with childhood recurrent infections, progressive muscle weakness, and mild to moderate mental and behavioral deficits, usually seen in Arts syndrome. This myriad of symptoms shows that CMTX5 and Arts syndrome can overlap in patients expressing PRS-I hypoactivity, supporting the theme of a continuous spectrum of phenotypes [21].

The overlapping phenotype exhibited by the p.Q277P mutation can also be shown by the affect it has on PRS-I's quaternary structure. Located at the trimer interface, this mutation affects the catalytic site (similar to other Art syndromes causing mutations), mainly R5P binding site, but does not affect the allosteric sites (similar to CMTX5) [21].

## 8. Arts Syndrome

The most severe form of X-linked PRS-I hypofunction results in Arts syndrome. This syndrome is characterized by symptoms that all appear before two years of age, including profound bilateral sensorineural hearing loss, hypotonia, delayed motor development, intellectual disability, ataxia, and increase risk of infection (upper respiratory tract). Peripheral neuropathy and optic atrophy also occur in early childhood. Unfortunately, this disease is lethal with 80% of reported patients with Arts syndrome dying before the age of six [3, 39].

Two loss-of-function* PRPS1* mutations have been associated with Arts syndrome, namely, c.398A>C (p.Q133P) and c.455T>C (p.L152P) [22]. These mutations impair PRS-I activity most severely, thereby resulting in the most severe form of PRS-I related disorders. The p.Q113P change is predicted to destabilize the homodimer at the dimer interface (allosteric site II) and to severely destabilize the ATP binding pocket [22]. The p.L152P substitution causes interatomic interference that destabilizes the structure predominantly affecting the ATP binding site and hence PRS-I activity [22].* In vitro* enzymatic activity assays in affected patients' cultured fibroblasts have a 13-fold decrease in PRS-I activity compared to controls [5].

## 9. Recent Novel* PRPS1* Mutation

Recently, a novel phenotype has been associated with* PRPS1* missense mutation, c.586C>T (p.R196W), in two male siblings leading to decreased PRS-I function [23]. The patients showed prenatal high *α*-fetoprotein (MS-AFP), intrauterine growth restriction, dysmorphic facial features, central nervous system abnormalities, white matter changes, severe intellectual disability, and spastic quadraparesis. These patients also developed Leber's congenital amaurosis and short stature. For the first time,* PRPS1 *deficiency was associated with diabetes insipidus. This unusual phenotype expands the spectrum of* PRPS1-*related diseases and demonstrates the crucial role of* PRPS1* in nervous system development. The p.R196W mutation affects the interaction of PRS-I with pyrophosphate and destabilizes the transition state resulting in decreased enzyme activity [23]. The identification of additional patients having this* PRPS1 *mutation will lead to the better characterization of phenotype-genotype correlation.

## 10. Gain-of-Function Treatment Options

Excess purine production results in hyperuricemia and hyperuricosuria, ultimately turning into uric acid and subsequently gouty arthritis, if left untreated. This is the classic finding for patients with PRS-I superactivity. The main goal of therapy for these patients is to lower the concentration of purine nucleotides and uric acid production and to prevent or even reverse urate crystal deposition. Treatment requires a dietary change and supplemental medication to be effective. Patients diagnosed with PRS-I superactivity should avoid red meat, shellfish, oily fish, and high-fructose corn syrup while increasing their low-fat dairy intake. In addition, prescribed allopurinol will help decrease uric acid production. Allopurinol is a xanthine oxidase inhibitor, stopping the conversion of oxypurine hypoxanthine and xanthine to uric acid; it also decreases* de novo* synthesis of purine nucleotides [39].

## 11. Loss-of-Function Treatment Options

To counter the decreased concentrations of crucial purine nucleotides, as seen with PRS-I loss-of-function disorders, it is necessary to find a method to replenish the nucleotides. Unlike dietary pyrimidines, which cross the intestinal barrier unharmed, dietary purine nucleotides are usually oxidized to uric acid by intestinal enzymes. However, dietary S-adenosylmethionine (SAM) freely crosses both the intestinal and the blood brain barrier. In an alternate pathway, independent of PRPP, SAM can theoretically replenish GTP and ATP (Figure 1). There are two pathways in the human body in which SAM can be converted into adenosine and indirectly to GTP; methyltransferases convert SAM into S-adenosylhomocysteine, which is then converted into adenosine by S-adenosylhomocysteine hydrolase, and secondly SAM is converted directly into adenosine via the polyamine pathway (Figure 1). Adenosine can then be converted into AMP via adenine phosphoribosyltransferase (APRT). AMP can be converted into IMP via AMP deaminase, which subsequently can be turned into GTP. SAM treatment has been used to treat 2 patients with Arts syndrome (J Christodoulou et al. unpublished data) and patients with hypoxanthine phosphoribosyltransferase (HPRT) deficiency [40]. It has also been shown to elevate previously low purine nucleotide levels, alleviating the clinical symptoms, and is shown to slow the progression of hearing impairment. With early detection and diagnosis, SAM supplementation is proven to be an effective therapy in slowing down the progression and onset of both neurological and audiologic symptoms in patients with Arts syndrome. Studies are warranted for the effectiveness of SAM supplementation with less severe forms of loss-of-function PRS-I mutations.

## 12. Conclusion

PRS-I plays a crucial role in human body by producing the necessary purine and pyrimidine nucleotides. Gain-of-function and loss-of-function mutations in* PRPS1 *have been found and can result in a wide spectrum disorders with overlapping phenotypes depending on the residual capacity of the PRS-I enzyme. These gene mutations manifest as either altering one of the two allosteric regulation sites (gain of function) or destabilizing the active binding site (loss of function) on the PRS-I enzyme. On one end of the spectrum, increase in PRS-I activity results in PRS-I superactivity with its defining characteristic of childhood gout and variable symptoms of sensorineural hearing loss, hypotonia, and ataxia depending on the severity of the mutation. On the other end, loss of function can result in a spectrum of phenotypes from mild to severe: DFN-2, CMTX5, and Arts syndrome. Decreased PRS-I activity is predominantly characterized by sensorineural hearing loss with variable symptoms of optic atrophy, ataxia, peripheral neuropathy, delayed motor development, and intellectual disability. Currently, treatment options for PRS-I related disorders are limited and not curative and only help slow the progression of the disease. For patients with PRS-I gain-of-function mutations resulting in uric acid overproduction, dietary restrictions and allopurinol are the mainstay treatment options. For loss-of-function mutations resulting in sensorineural hearing loss, SAM supplements in patients with Arts syndrome are able to replenish ATP and GTP concentrations to some extent, therefore slowing the progression of sensorineural hearing loss, and alleviate some of the neurological symptoms. Further large scale studies and clinical trials will help in assessing the therapeutic potential of SAM supplementation in alleviating symptoms due to* PRPS1* mutations. Identification of novel biomarkers and detection of manifestations at the prenatal stage will allow the initiation of therapeutic intervention early in the disease course with improved outcomes.

## Figures and Tables

**Table 1:** Mutations identified in *PRPS1* leading to altered enzyme function.

Disorder	Gene Mutation	Amino Acid Change	Effect on *PRPS1 * Function	Reference
PRS-1 Superactivity	c.154G>C	p.D52H	Gain of Function	Becker et al. [13]
PRS-1 Superactivity	c.385C>A	p.L129I	Gain of Function	Becker et al. [13]
PRS-1 Superactivity	c.341A>G	p.N114S	Gain of Function	Roessler et al. [14]
PRS-1 Superactivity	c.547G>C	p.D182H	Gain of Function	Roessler et al. [14]
PRS-1 Superactivity	c.569C>T	p.A189V	Gain of Function	Becker et al. [15]
PRS-1 Superactivity	c.578A>T	p.H192L	Gain of Function	García-Pavía et al. [16]
PRS-1 Superactivity	c.579C>G	p.H192Q	Gain of Function	Becker et al. [13]
PRS-1 Superactivity and Arts Syndrome	c.424G>C	p.V142L	Gain of Function	Moran et al. [17]
DFN2	c.193G>A	p.D65N	Loss of Function	Liu et al. [5]
DFN2	c.259G>A	p.A87T	Loss of Function	Liu et al. [5]
DFN2	c.869T>C	p.I290T	Loss of Function	Liu et al. [5]
DFN2	c.916G>A	p.G306R	Loss of Function	Liu et al. [5]
DFN2 and CMTX5	c.337G>T	p.All3S	Loss of Function	Robusto et al. [18]
DFN2 and CMTX6	c.343A>G	p.M115V	Loss of Function	Robusto et al. [18]
DFN2 and CMTX7	c.925G>T	p.V309F	Loss of Function	Robusto et al. [18]
DFN2 and CMTX8	c.362C>G	p.A121G	Loss of Function	Park et al. [19]
CMTX5	c.129A>C	p.E43D	Loss of Function	Kim et al. [20]
CMTX5	c.344T>C	p.M115T	Loss of Function	Kim et al. [20]
CMTX5 and Arts syndrome	c.830A>C	p.Q277P	Loss of Function	Synofzik et al. [21]
Arts Syndrome	c.398A>C	p.Q133P	Loss of Function	de Brouwer et al. [22]
Arts Syndrome	c.455T>C	p.L152P	Loss of Function	de Brouwer et al. [22]
Arts Syndrome	c.856C>T	p.R196W	Loss of Function	Al-Maawali et al. [23]
